# Dynamic Time Warping Algorithm in Modeling Systemic Risk in the European Insurance Sector

**DOI:** 10.3390/e23081022

**Published:** 2021-08-08

**Authors:** Anna Denkowska, Stanisław Wanat

**Affiliations:** Department of Mathematics, Cracow University of Economics, ul. Rakowicka 27, 31-510 Kraków, Poland

**Keywords:** time series analysis, minimum spanning trees, topological indicators of the MST, dynamic times warping, insurance sector, systemic risk, G22, C10

## Abstract

We are looking for tools to identify, model, and measure systemic risk in the insurance sector. To this aim, we investigated the possibilities of using the Dynamic Time Warping (DTW) algorithm in two ways. The first way of using DTW is to assess the suitability of the Minimum Spanning Trees’ (MST) topological indicators, which were constructed based on the tail dependence coefficients determined by the copula-DCC-GARCH model in order to establish the links between insurance companies in the context of potential shock contagion. The second way consists of using the DTW algorithm to group institutions by the similarity of their contribution to systemic risk, as expressed by DeltaCoVaR, in the periods distinguished. For the crises and the normal states identified during the period 2005–2019 in Europe, we analyzed the similarity of the time series of the topological indicators of MST, constructed for 38 European insurance institutions. The results obtained confirm the effectiveness of MST topological indicators for systemic risk identification and the evaluation of indirect links between insurance institutions.

## 1. Introduction

The European Insurance and Occupational Pensions Authority (EIOPA) [1] acts as an independent advisor to the European Parliament. In a document from 2017, it noted that the dynamics of mutual connections in the insurance sector has significant importance. It was indicated that the phenomenon of systemic risk should be particularly analyzed in the context of contagion, i.e., the spread of negative effects of financial disturbances in the sector. The aim of this paper was to investigate the applicability of network theory tools in conjunction with the Dynamic Time Warping algorithm.

We used the Dynamic Time Warping (DTW) algorithm in two ways to evaluate the suitability of Minimum Spanning Trees’ topological indicators in the context of systemic risk (SR) and to construct the MST, to establish the similarity between the time series of the DeltaCoVaR measure. We showed the contribution of the surveyed insurance institutions to the systemic risk of the insurance sector in the different market states. In the paper, we analyzed the dynamics of indirect connections between insurance companies that result from market price channels. In that analysis, we assumed that the stock quotations of insurance companies reflect market sentiments that constitute a very important systemic risk factor. Interlinkages between insurers and their dynamics have a direct impact on systemic risk contagion in the insurance sector. We proposed as in [2] “a hybrid approach to the analysis of interlinkages dynamics based on combining the copula-DCC-GARCH model and Minimum Spanning Trees (MST).”

A Minimum Spanning Tree is a connected and acyclic graph with the smallest sum of weights assigned to each edge, where each vertex is an insurance institution and the edges connect those lying at relatively small distances, i.e., those companies that share important similarities. Using the copula-DCC-GARCH model we determined the tail dependence coefficients. Then, for each analysed period t for (t=1,…,T) we constructed $MST_{t}$ with k vertices and k−1 edges based on these coefficients using the Kruskal algorithm. The graph $MST_{t}$ models the state of the network of connections between insurance companies in the period t. The vertices of the graph are individual insurance companies, and edges indicate the existence of relatively small distances and thus similarities between pairs of insurance companies. $MST_{t}$ can be considered filtered networks, which allow us to identify the most likely and the shortest crisis (systemic risk) transmission path. In the analyzed period, indirect connections between insurance companies that result from market price channels change; therefore, the $MST_{t}$ reflecting them is characterized by a different structure. We can distinguish a structure resembling a star or a chain. The star structure indicates the connections between institutions that, in the event of negative financial events, will be the transmission path of the negative effects of these events. Such a structure is associated with the unwanted effect of infection (risk propagation). The MST chain structure shows that there are fewer links between institutions. The transmission of the effects of disturbances or the disturbances themselves are dissipated more slowly if MST is more diffused.

Thus, by observing the MST topology, we can assess the links between institutions in the context of the possibility of propagating systemic risk. To assess the topological structure of MST, we used selected topological indicators in the years 2005–2019. The times series obtained from the proposed hybrid approach reflect the phenomena occurring on the market. We answered the question of whether the analyzed MST topological indicators can be considered to identify and model systemic risk. We used the DTW algorithm, which allows one to determine the similarity between time series, which may be of different length and are distorted (stretched or shifted) in relation to the time axis. We established the similarity of time series in periods of financial crises and in periods outside of crises. The similarity of time series in the respective states confirmed that the topological indicators of MST constructed in this way are a tool that allows the identification of SR in the insurance sector and reflects the dynamics of mutual indirect interrelations between insurers.

Moreover, in this paper, we examined the contribution of a single insurer to the systemic risk of the European insurance sector using the measure DeltaCoVaR. The CoVaR measure is the basis for measuring SR. Formally, in [3], $CoVaR^{j|i}{}_{\beta ,t}$ is defined as the value at risk at the β significance level (VaR) of an institution j under the condition that another institution i is at risk of crisis in a given period t. Then, for the second time in this paper, we used the DTW algorithm in a different context. This time, we established DTW (i,j)—the similarity of the DeltaCoVaR time series between pairs (i,j) of insurers in periods of different crises and in the normal period. Then, based on the DTW (i,j) distance matrix, we constructed the MST for these periods, and thus, we grouped insurance institutions according to the similar contribution to systemic risk, i.e., the DeltaCoVaR measure.

Systemic risk in the insurance sector has attracted much attention lately. It has been studied by: [4,5,6,7,8,9,10] and risk infection has been studied by [11,12,13]; nevertheless, none of these approaches are a hybrid approach in which the possibility of combining different measures would be analyzed as proposed in our project. Dynamic Time Warping (DTW) is one of the algorithms for measuring the similarity between two time series of different length, which may differ with time. The algorithm itself [14] was introduced in the 1960s. In recent years, Dynamic Time Warping (DTW) has emerged as the distance measure of choice for virtually all time series data mining applications. DTW is so far a widely used algorithm in noneconomic fields such as speech recognition [15], image [16] or motion recognition [17], ECG analysis, biometrics, signal analysis, and data mining. The paper [18] showed that the nonparametric DTW measure of similarity is better than other measures, such as the Pearson correlation coefficient, and yet [19] stated that economic research has not yet used the full potential of Dynamic Time Warping. We can also find studies in the literature in economic research that have used the DTW technique [20,21]. MST is widely used in financial network analysis. Examples may be the works in which the topological indices of MST we are analyzing have also been examined by [22,23,24,25].

## 2. Data and Methodology

The study was based on the stock quotes of 38 European insurance institutions. Most of them can be found among the top 50 insurance companies in Europe based on total assets. These are: “Achmea (Eureko Group), Aegon Group/Unirobe Meeùs Group, AGEAS, Allianz, Aviva, AXA, BNP Paribas, Grupo Catalana Occidente, CNP Assurances, Royal Bank of Scotland Group, Generali, Groupe Crédit Agricole Assurances, HDI/Talanx, If P&C Insurance, ING Group, KBC, Legal & General Group plc, Mapfre, Munich Re, Old Mutual plc, Prudential, RSA Insurance Group, SCOR, Lloyds Banking Group, Unipol, UNIQA Insurance Group, Vienna Insurance Group, Zurich Insurance, Swiss Life, Chubb Ltd., Hannover Re, Storebrand, XL.Group, Helvetia Holding, Mediolanum, Sampo Oyj, Societa Cattolica di Assicurazione, and Topdanmark A/S.” We analyzed weekly logarithmic returns for the period from 7 January 2005 to 20 December 2019.

### 2.1. MST Construction Based on the Tail Dependencies of the Log-Returns

As in the paper [2], we analyzed changes in interconnections in the European insurance sector using a new methodology. For the construction of Minimum Spanning Trees (MST), we used the relationships in the tails of logarithmic rates of return, estimated with the use of the copula-DCC-GARCH model. Therefore, based on two-dimensional copula-DCC-GARCH models for each studied period t, (t=1,…,T) and each pair of rates of return $r_{i,t},\;r_{j,t}$, (i,j=1,…,k, j>i), we estimated the bivariate joint distributions:

(1) $$F_{t}\left(r_{i,t},r_{j,t}\right)=C_{ij,t}\left(F_{i,t}\left(r_{j,t}\right),F_{j,t}\left(r_{i,t}\right)\right)$$

where $C_{ij,t}$ is the copula, and $F_{t}$ and $F_{i,t}$, $F_{j,t}$, respectively, denote the joint cumulative distribution function and the cumulative distribution functions of the marginal distributions at time t. Further on, using the copulas $C_{ij,t}$ we estimated the pairwise lower tail dependence of the returns $r_{i,t},\;r_{j,t}$:

(2) $$\lambda_{t}^{L}\left(i,j\right)=\underset{q\to 0^{+}}{\lim}\frac{C_{ij,t}\left(q,q\right)}{q}.$$

We considered various possible copula-DCC-GARCH two-dimensional models to estimate $\lambda_{t}^{L}\;\left(i,j\right)$. For all instruments, we assumed the ARMA (1,1) -sGARCH (1,1) model with the Student skew distribution. However, to examine the dynamics of the relationship between log-returns, we used Student copulas with correlations established using the DCC (1, 1) model and a constant shape parameter. We selected the above specifications on the basis of information criteria and appropriate model adequacy tests.

Then, for each period *t*, we determined the “distance” matrix between insurance companies using the metric (Mantegna and Stanley, 1999):

(3) $$d_{t}\left(i,j\right)=\sqrt{2\left(1-\lambda_{t}^{L}\left(i,j\right)\right)}$$

and using the Kruskal algorithm (Mantegna and Stanley, 1999), we constructed Minimum Spanning Trees $MST_{t}$ with k vertices and k−1 edges.

### 2.2. MST Topological Indicators

Based on the trees thus obtained, $MST_{t}$ $\left(t=1\ldots T\right)$, we determined the time series of the following topological network indicators, whose definitions and interpretations in the context of systemic risk can be found in the work [2]:

- Average Path Length (APL) [25]. For MST, it is an indicator that tells you the average number of steps between all possible pairs of vertices in the network. As it is an average number of steps, the network may well have several vertices separated by a large number of steps and many others lying close to each other, i.e., needing few steps. This indicator is a measure of the effectiveness of the information flow. It is seen in the literature as one of the strongest measures of MST structure. It distinguishes between easy-to-transition networks and those that are complex and inefficient. The structure of the network of interconnections between financial institutions is important for systemic risk. This indicator tells you whether the MST data have a structure that will be vulnerable to infection with adverse effects of negative financial events. Or, on the contrary, as a network of difficult and distant connections, it will slow down the flow of information.
- Network Diameter (Diameter) [23]. This is an MST indicator obtained as the length of the longest geodesic path between any two nodes. This distance is determined as the sum of the weights assigned to the edges connecting the vertices. If the Diameter shrinks, it means the distance between the vertices lying most apart is decreasing. This network structure favors the transfer of information.
- Maximum Degree (Max.Degree) [25]. The degree of an MST vertex is the number of its connections to adjacent vertices. After determining the vertex that has the greatest number of such connections, this number is assigned to MST as the Maximum Degree. Any dynamic change of the Maximum Degree alters the structure of the network. The increase in Maximum Degree means that there is a vertex in the network that has numerous connections to other vertices. For systemic risk, the MST structure with a high Max.Degree offers the possibility of rapid shock contagion.
- Parameter “alpha” of the power law of the degree distribution [25]. This is a parameter of a power distribution of vertex degrees. For each MST, it can be checked whether the vertex distribution is consistent with the power distribution and has the form P (s) = C ∙ s ^ (−α), α > 0, for α, which is a parameter related to a given MST. If the α parameter belongs to the range (2,3), it means that the network structure is scale-free and has self-similarities, i.e., a repeating structure. When analyzing SR, such MSTs with hub nodes can promote the spread of negative financial information. Hub vertices, i.e., institutions that have numerous connections, are potentially systemically important institutions. If such an institution is threatened, the consequences of this situation could be felt by many related financial institutions.
- Rich Club Effect (RCE) [22,23] specifies how the vertices are connected. The RCE for a fixed vertex degree k tells you how many times the vertices of degree k are connected to those of degree k or higher. RCE determines the stability of the network. The higher the RCE, the easier the information is conveyed. The structure of the MST is more compact for higher RCE, which potentially enables contagion and transferability of the negative effects of shocks in the financial market.
- Assortativity [24] evaluates the entire MST structure, how vertices in the network are generally linked one to another: whether the high-degree vertices connect with those that also have a high degree, or with low-degree ones. Assortativity, expressed with scalar ρ, takes values in the range (−1,1). Negative assortativity means that high-degree vertices merge into low-degree vertices. Positive assortativity means that high-degree vertices converge into high-degree vertices. Assortativity informs about the structure of the network and its dynamic behavior, as well as resistance to the random spread of the crisis.

MST can reveal the roles of individual stocks. MST topological indicators, such as Betweenness Centrality, Vertex degree, Vertex strength, and Closeness Centrality, allow the assessment of the role and importance of a single institution in the entire network. Nevertheless, this area was not addressed in this study.

### 2.3. Market Regimes under Study

In this paper, for the mentioned MST topological indicators, we determined the DTW distance between the series in the following periods:

- Subprime Mortgage Crisis (SMC): the period of two subprime crises and excessive public debt, which began in 2008 and lasted until around 2013. This period in our time series stretches exactly between 8 February 2008 and 1 March 2013
- Immigrant (I): the period of crisis associated with the beginning of the migration crisis in Europe in 2015/2016. This period on our time series stretches exactly from 7 August 2015 to 23 September 2016
- France and Italy Crisis (FIC): crisis in the countries of the European Union related to the crisis in France associated with strikes, and in Italy due to the ever-growing public debt (which is now seven times higher than the debt in Greece), falling at the turn of 2017 and 2018. In our case, it is exactly the period from 21 April 2017 to 11 May 2018
- Normal (N), which is set in the remaining time intervals. It consists of four periods: $N_{1}$ (7 January 2005–1 February 2008), $N_{2}$ (8 March 2013–31 July 2015), $N_{3}$ (30 September 2016–14 April 2017), and $N_{4}$ (18 May 2018–20 December 2019).

### 2.4. Dynamic Time Warping Algorithm

For each topological indicator, we analyzed the similarity of the time series in the periods mentioned, applying the principle of comparing each period with each other period. We treated given episodes of series as time series of various lengths $X=\left(x_{1},\ldots ,x_{n}\right)\;\mathrm{and}\;Y=\left(y_{1},\ldots ,y_{m}\right)\;\mathrm{for}\;n\;\left\{\;1,\;2\;,\;\ldots ,\;N\;\right\},\;m\;\left\{1,\;2\;,\ldots \;,\;M\;\right\},$ with N, MN. The DTW algorithm establishes the local measure of costs, sometimes called the local distance measure, i.e., a function 𝜃$:S\times S\to R_{+}.$ Usually, $\theta \left(x,y\right)$ is small (low cost) if *x* and *y* are similar; otherwise, $\theta \left(x,y\right)$ is large (high cost). Determining the local cost measure for each pair of terms of *X* and *Y* series, we obtained a cost matrix $\Theta_{N\times M}$ defined by Θ$\left(n,m\right)≔\theta \left(x_{n},y_{m}\right).$ The local cost measure was determined by 𝜃$\left(x_{n},y_{m}\right)={\left(x_{n}-y_{m}\right)}^{2}$. Then, the warping path was determined. A warping path is a sequence $w=(w_{1},\ldots ,\;w_{K}),$ for $w_{l}=\left(n_{l},m_{l}\right)\in \left\{1,\ldots ,N\right\}\times \left\{1,\ldots ,M\right\},for\;k\in \left\{1,\ldots ,K\right\}$ and meets the following conditions:

- Boundary condition: $w_{1}=\left(1,1\right)\;\mathrm{and}\;w_{K}=\left(N,M\right).$
- Monotonicity condition: $n_{1}\;\leq \;n_{2}\;\leq \;\ldots \;\leq \;n_{K}\;\mathrm{and}\;m_{1}\;\leq \;m_{2}\;\leq \;\ldots \;\leq \;m_{K}.$
- Step size condition: $w_{k+1}-w_{k}\in \;\left\{\left(1,\;0\right),\left(0,\;1\right),\left(1,\;1\right)\right\}\;\mathrm{for}\;k\in \left\{1,\ldots ,\;K-1\right\}$.

The total cost $\theta_{w}\left(X,Y\right)$ of the *warping path* w between *X* and *Y* with respect to the local cost measure $\theta \left(x,y\right)$ is defined as $\theta_{w}\left(X,Y\right)≔\sum_{k=1}^{K}\theta \left(x_{n}{}_{k},y_{m}{}_{k}\right)$. An optimal warping path between *X* and *Y* is denoted by $w^{*}$—it is a warping path with the minimum total cost of all possible paths.

(4) $$DTW\left(X,Y\right)≔\theta_{w^{*}}\;\left(X,Y\right)\;=\min\left\{\theta_{w}\left(X,Y\right),\;where\;w\;is\;an\;\left(N,M\right)\;warping\;path\right\}$$

The normalized *DTW* distance is the distance divided by *N* + *M*, where *N* and *M* are the lengths of the time series *X* and *Y*, respectively.

### 2.5. DeltaCoVaR Measure

By examining the contribution to systemic risk of all the analyzed insurance institutions, we established a standard DeltaCoVaR measure for each of them based on the estimation $CoVaR^{j|i}{}_{\beta ,t}$ described in the paper [3].

Measure $CoVaR^{j|i}{}_{\beta ,t}$ is the value at risk (VaR) of an institution j under the condition that another institution i is at risk of crisis in a given period t, i.e., its rate of return is less than its value at risk:

(5) $$P(r_{j,t}\leq CoVaR^{j|i}{}_{\beta ,t}|r_{i,t}\;\leq \;VaR^{i}{}_{\alpha ,t})=\beta$$

Using the formula for the conditional probability, we have:

(6) $$\frac{P\left(r_{j,t}\leq \;CoVaR^{j|i}{}_{\beta ,t},\;r_{i,t}\;\leq \;VaR^{i}{}_{\alpha ,t}\right)}{P\left(r_{i,t}\;\leq \;VaR^{i}{}_{\alpha ,t}\right)}=\beta$$

In addition, the definition of value-at-risk for institutions and implies that

(7) $$P\left(r_{i,t}\leq VaR^{i}{}_{\alpha ,t}\right)=\alpha$$

From Equations (6) and (7), we obtain:

(8) $$P(r_{j,t}\leq CoVaR^{j|i}{}_{\beta ,t},r_{i,t}\;\leq \;VaR^{i}{}_{\alpha ,t})=\alpha \beta$$

From (8), we can estimate $CoVaR^{j|i}{}_{\beta ,t}$ once we have determined the two-dimensional distribution $F_{t}$ of the rate-of-return vector $(r_{j,t,\;}r_{i,t}).$ This distribution can be represented using the copula in the following ways:

(9) $$F_{t}(r_{j,t,\;}r_{i,t})=C_{t}\left(F\left(r_{j,t}\right),F\left(r_{i,t}\right)\right)$$

From Formula (9), we can numerically calculate $CoVaR^{j|i}{}_{\beta ,t}$ by solving the equation:

(10) $$C_{t}\left(F_{t}\left(CoVaR^{j|i}{}_{\beta ,t}\right),\alpha \right)=\alpha \beta$$

The value of the measure $CoVaR^{j|i}{}_{\beta ,t}$ permits the computation of the measure DeltaCoVaR ($\Delta CoVaR_{\beta }{}^{j|i}$), which is the difference between the value at risk of the insurance sector (institution j), provided that the insurer (institution i) is in a state of financial crisis and the value at risk of the insurance sector when the financial standing of the entity i is normal (average), i.e.,

(11) $$\Delta CoVaR_{\beta }{}^{j|i}=CoVaR_{\beta }{}^{j|X^{i}=VaR^{i}{}_{\alpha }}-CoVaR_{\beta }{}^{j|X^{i}=Median^{i}}$$

The value of this measure represents the institution’s share in generating systemic risk. In order to estimate the DeltaCoVaR measure, we assumed that the European insurance sector is represented by the STOXX 600 Europe Insurance index.

## 3. Empirical Results and Discussion

### 3.1. MST’s Topological Indicators in the Determined Market Regimes

**Figure 1:**
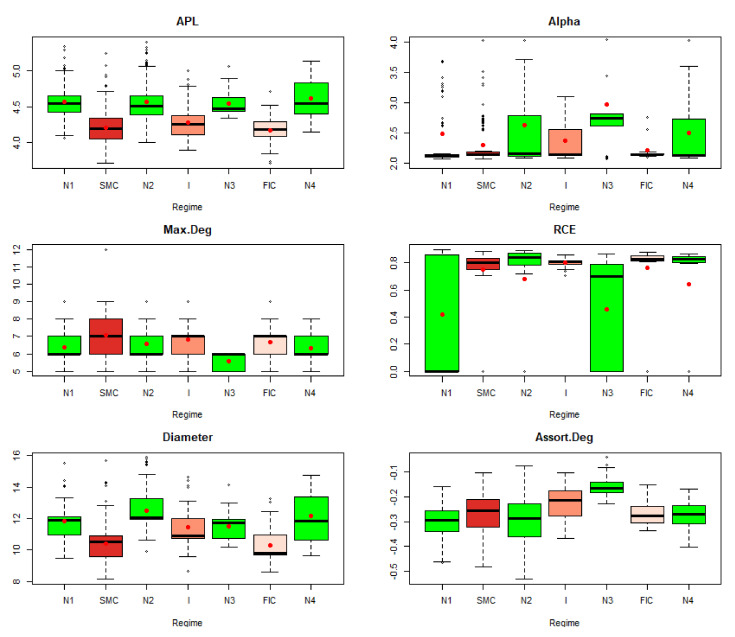
MST’s topological indicators in the periods under study. Own source.

MST’s topological indicators constructed based on the dependencies in the tails present different behaviors in the distinguished market states. Figure 1 shows boxplots with marked average values of indicators presented. The analysis shows that during crises, MSTs shrink, as evidenced by the decreasing APL and Diameter and the growing Max.Deg, which is favorable to the potential spread of undesirable effects of the shocks on the insurance market. MSTs are scale-free in the studied period, because the alpha parameter in the tested states belongs to the interval (2,3). The mean RCE for *k* = 4, where k is the degree of the vertex, is on a similar level. Thus, the number of attached vertices with four or more connections does not increase. In the periods $N_{1}$, it drops to zero, and in the period $N_{3}$, it decreases as compared to other periods. MSTs are nonassortative according to the previous definition, as the numbers are negative throughout the period considered. The smaller the value, the more high-degree vertices are connected to low-degree vertices. During the SMC and FIC periods, assortativity is higher than in neighboring normal states. That is, high-degree vertices are attached to vertices with more connections than in neighboring normal states.

In the next part of the research, we wanted to check the similarity of the dynamics of the behavior of the time series of the analyzed topological indices. Thus, we wanted to confirm the possibility of using the proposed methodology for the identification, modeling, and measurement of systemic risk in the insurance sector.

### 3.2. Analysis of the Similarity of Time Series of Topological Indicators Using the DTW Algorithm

In the empirical analysis, we determined the normalized DTW between the time series of the topological indicators of MST in the periods established earlier. We analyzed the similarity of the series in different normal periods, in different crisis periods, and between different normal periods and different crisis periods, as previously described. The results obtained are shown in Table 1. When interpreting the results obtained, it is assumed that the smaller the value of the normalized DTW, the more similar the fragments of time series in the distinguished regimes. To make Table 1 more readable, we inform the reader that the interpretation of the similarity of time series for each MST topological index in fixed periods—DTW values—together with a description of these relationships is included in the following section.

We investigated the possibility of using the DTW algorithm to assess the similarity of the time series of MST topological indicators in the determined states. Each of the indicators has a different role and reflects a different property of MST. A set of many, appropriately selected indicators can allow us to assess the dynamics of the MST structure in the determined states. As each normal period and each crisis is characterized by their own dynamics, we expect the DTW values to be different. We checked, however, whether the time series of MST topological indices in the period of crises have different dynamics than in other periods, called Normal.

**Figure 2:**
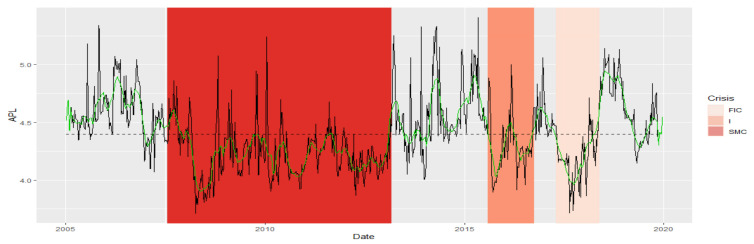
Average Path Length in the periods under study. Own source.

**Figure 3:**
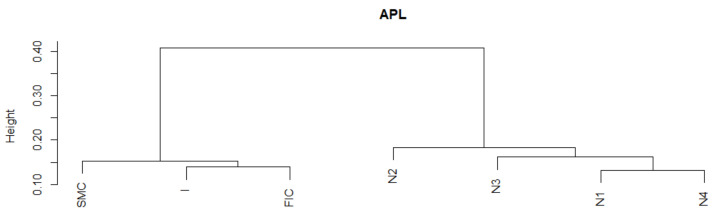
Dendrogram for APL-based hierarchical clustering of the periods under study. Own source.

Average Path Length (APL) is one of the most robust measures of network topology. The DTW results indicate a greater similarity of the APL time series fragments in the periods of SMC, I, FIC crises, and in normal periods. The established DTW normalized for pairs of $N_{i}-\mathrm{SMC}$, $N_{i}-I$, and $N_{i}-\mathrm{FIC}$ periods, for i=1,2,3,4 is much greater than for $N_{i}-N_{j}$ pairs consisting of normal periods only, or for pairs consisting of the crisis periods themselves of the APL time series (Table 1). The series are the least similar in the periods established between the pairs of $N_{i}-\mathrm{SMC}$, $N_{i}-I$, and $N_{i}-\mathrm{FIC}$ periods, for i=1,2,3,4. We know from Figure 1 that the average APL during the crises is lower than during the total regime of normal states, which means that during crises, the structure of MST favored the spread of the negative effects of financial turmoil. We present the similarity of a fragment of the series of the APL time series in particular periods using the cluster dendrogram developed using Ward’s method (Figure 2 and Figure 3).

**Figure 4:**
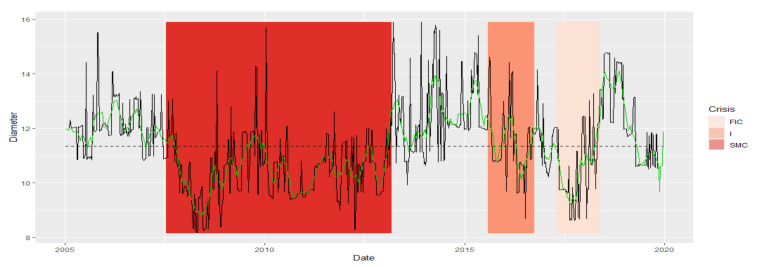
Diameter in the periods under study. Own source.

**Figure 5:**
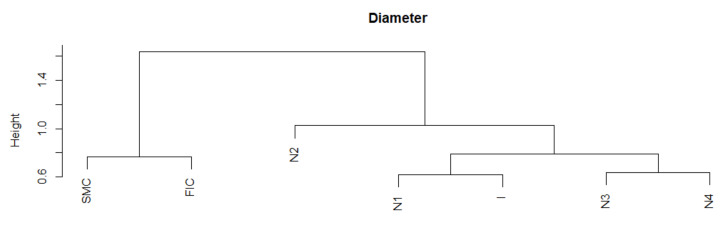
Dendrogram for Diameter-based hierarchical clustering of the periods under study. Own source.

The similarity of the Diameter series is analogous to the similarity of the APL series. There is a noticeable division into the group of SMC and FIC crises and a separate group of Normal states. Crisis I is in the group with Normal states. The series is the most similar during normal states. The largest of the SMC crises in comparison with the Normal periods is characterized by the least similarity with any fragment of the series (Table 1). Thus, Diameter MST during the SMC crisis has different dynamics than in other states. We present the similarity of a fragment of the series of the Diameter time series in particular periods using the cluster dendrogram developed using Ward’s method (Figure 4 and Figure 5).

The Max.Degree indicator remained at a similar level (Figure 1) during the crises. Now, we additionally receive information that the dynamics of the time series during SMC crises looks different for each crisis FIC or I. The series in normal periods $N_{1}$ and $N_{2}$ has the most similar fragments. The $N_{3}$ period stands out, most likely due to the shortest section of this series lasting only six months, and it is the least similar to any period. Moreover, a fragment of the series for SMC is distinguished, which is the least similar to the series fragments in the remaining periods. During the FIC and I crises, the time series is the most similar. Let us summarize the results of the DTW study for Max.Degree with the graph in Figure 1. The average Max.Degree (red dots in Figure 1) is the same during crisis periods, while the average Max.Degree is lower in adjacent normal periods. This means that in times of crisis, MST has an institution that has more connections than in normal times

**Figure 6:**
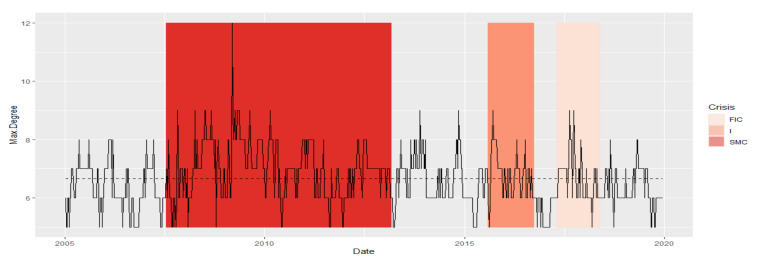
Max.Degree in the periods under study. Own source.

**Figure 7:**
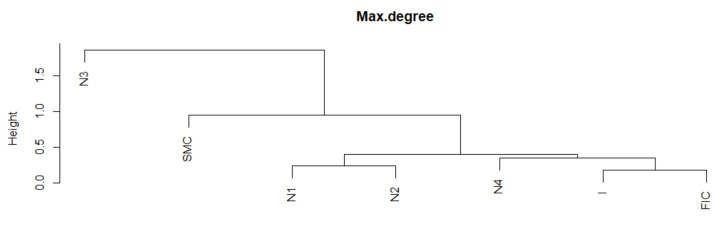
Dendrogram for Max.Degree-based hierarchical clustering of the periods under study. Own source.

We present the similarity of a fragment of the series of the Max.Degree time series in particular periods using the cluster dendrogram developed using Ward’s method (Figure 6 and Figure 7). Based on this dendrogram, we can say that this indicator in period I and FIC has similar dynamics. Although the average in the SMC period is similar, the dynamics of the change in this indicator is different than in the periods I and FIC, just as was the dynamics of the development of crises.

Due to the fact that during the entire period 2005–2019, MSTs are scale-free, as alpha has values in the range (2, 3), the similarity of the shape of this indicator series does not provide new information. Note that the period N3 lags behind, the periods FIC and I again are similar, and SMC is in the group with N4. The parameter alpha informs us about the scale-free structure of the MST. If alpha is in the interval (2, 3), then the corresponding MST is fractal-like, with institutions that are hubs. Now, we summarize the results of the DTW test for the alpha parameter with the graph in Figure 1. Analogically to Max.Degree, the mean alpha values in periods of crisis are different from those in normal periods. At the same time during crises, they are closer to 2, while in the neighboring periods, the averages of the alpha parameter are closer to 3, which means that the structure of MST during crises is more fractal (self-similar) than in normal periods.

**Figure 8:**
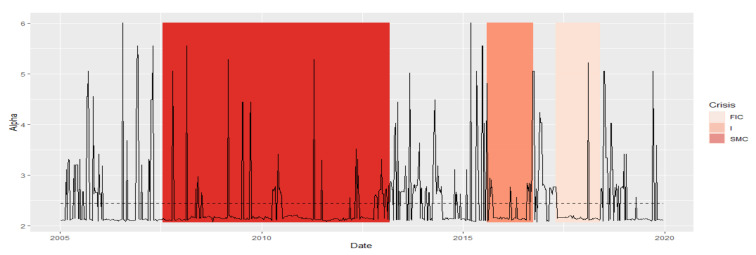
Alpha in the periods under study. Own source.

**Figure 9:**
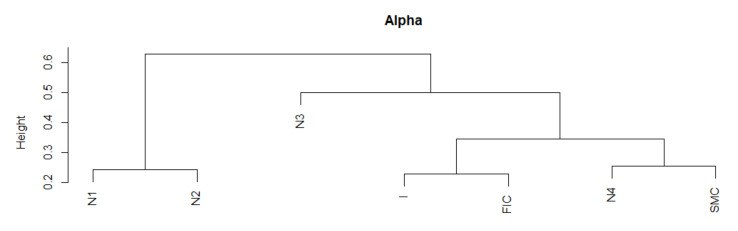
Dendrogram for alpha-based hierarchical clustering of the periods under study. Own source.

We present the similarity of a fragment of the series of the parameter alpha time series in particular periods using the cluster dendrogram developed using Ward’s method (Figure 8 and Figure 9).

The RCE for the k=4 series has variable dynamics. There is a similarity in the period of the greatest SMC crisis to the normal states $N_{4}$ and $N_{2}$. The remaining crises are in other groups in which the MST structures are similar in terms of the way they connect. Let us summarize the results of the DTW study for the RCE plotted in Figure 1. We see again in Figure 1 that the average RCE during crisis is at a significantly different level than in normal states. More specifically, during crises, the RCE average has higher values. For k = 4, it means that during the crisis, the number of insurance institutions with more than four connections with other institutions grows, which confirms that, during crises, the structure of MST becomes more star-like, which potentially creates the possibility of contagion and, thus, increases systemic risk.

**Figure 10:**
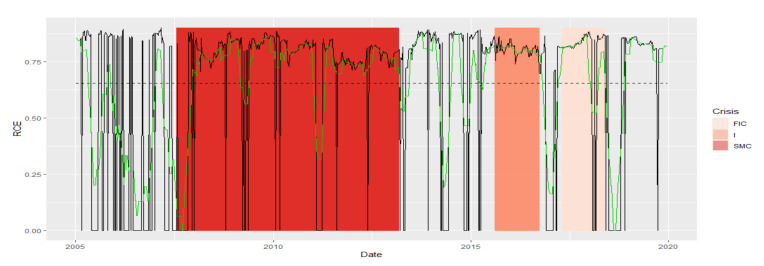
RCE in the periods under study. Own source.

**Figure 11:**
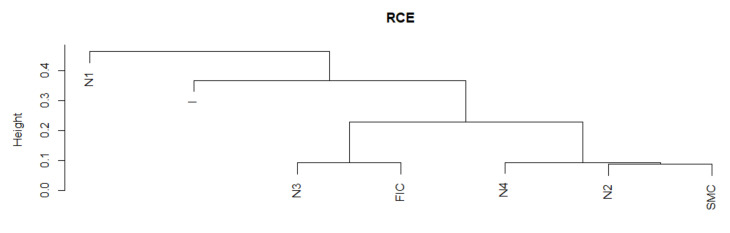
Dendrogram for RCE-based hierarchical clustering of the periods under study. Own source.

We present the similarity of a fragment of the series of the RCE time series in particular periods using the cluster dendrogram developed using Ward’s method (Figure 10 and Figure 11).

MSTs are not assortative in the entire analyzed period 2005–2019. Thus, the dynamics of this series are very similar throughout this period. A network is said to be nonassortative when high-degree vertices are connected to low-degree ones, while low-degree vertices are connected to high-degree ones. This negative mating means that vertices with high degrees associate with those that have low degrees, which goes hand in hand with the previously described network scale-free property. As a matter of fact, it is the only topological index of MST studied by us, for which the DTW did not resolve the similarity as expected, although the period I and FIC appeared in one group. In subsequent studies, its role shall be analyzed in more detail, and perhaps the method we use for such an exception will not work. Perhaps it is related to the fact that the group of the examined institutions is small. The authors will investigate this issue.

**Figure 12:**
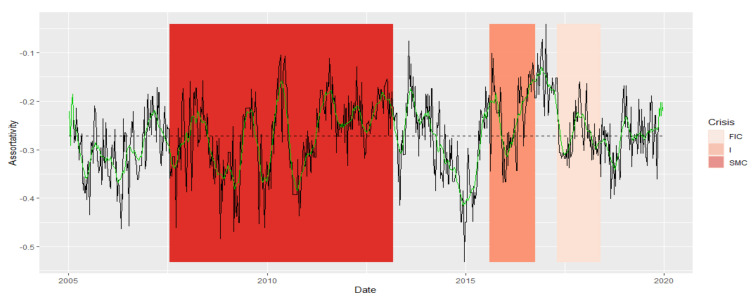
Assortativity in the periods under study. Own source.

**Figure 13:**
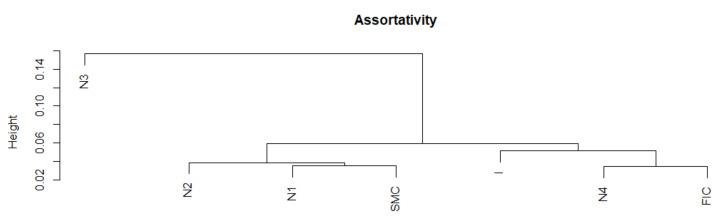
Dendrogram for assortativity-based hierarchical clustering of the periods under study. Own source.

We present the similarity of a fragment of the series of the assortativity time series in particular periods using the cluster dendrogram developed using Ward’s method (Figure 12 and Figure 13).

### 3.3. Analysis of the Mean DeltaCoVaR Time Series

**Figure 14:**
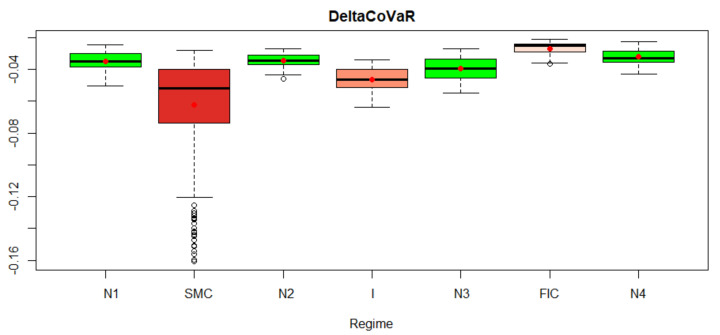
DeltaCoVaR measure for the analyzed market states. Own source.

In Figure 14, boxplots of the DeltaCoVaR measure for the analyzed market states are presented. The lower the DeltaCoVaR value, the greater the contribution of the studied institutions to the systemic risk of the insurance sector. The analysis shows that this contribution was greatest during the SMC and FIC crises.

**Figure 15:**
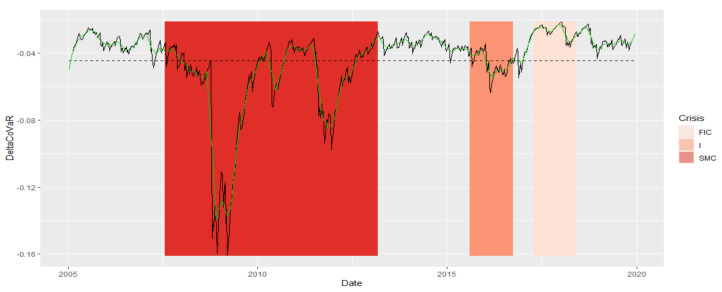
Average DeltaCoVaR for all analyzed institutions in the periods under study. Own source.

**Figure 16:**
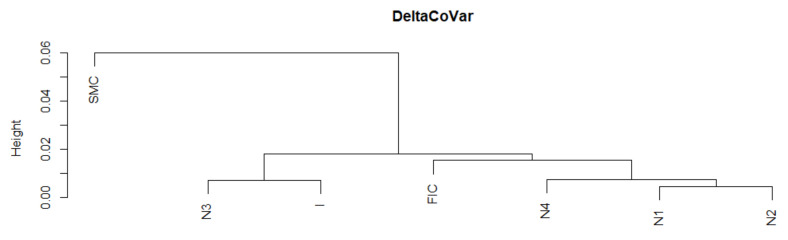
Dendrogram for DeltaCoVaR-based hierarchical clustering of the periods under study. Own source.

Below in Figure 15, we present the average DeltaCoVaR for all analyzed institutions. We studied the similarity of a fragment of this time series from the SMC period to other periods of crises or normal periods. On the dendrogram in Figure 16, SMC it is in a separate group. Thus, not only is the size of the SR contribution observable on the basis of the time series itself, but also the dynamics of this contribution as assessed by the DTW is different. In addition, the FIC or I crises are outside the group of similarities with most normal periods.

### 3.4. MST Based on DTW

Now, using Kruskal’s algorithm, we constructed MSTs based on the DTW (i,j) distance matrix, which show the similarity of the DeltaCoVaR time series between pairs (i,j) of insurers in states SMC, I, FIC, and N.

**Figure 17:**
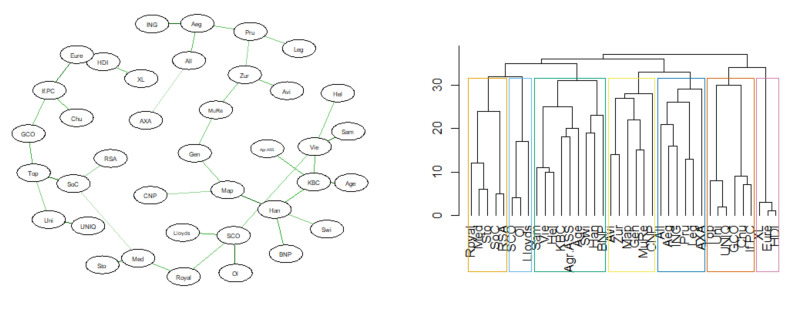
MST based on DTW in Normal state. Own source.

**Figure 18:**
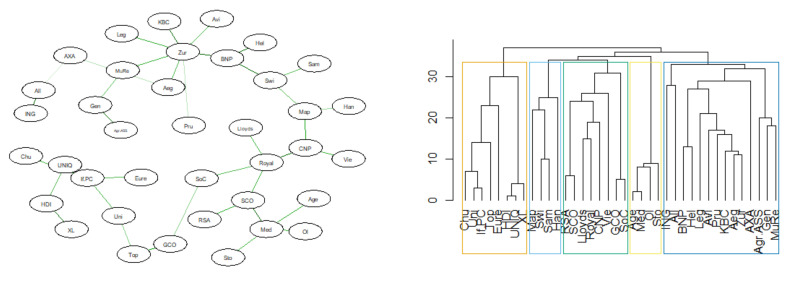
MST based on DTW in SMC state. Own source.

**Figure 19:**
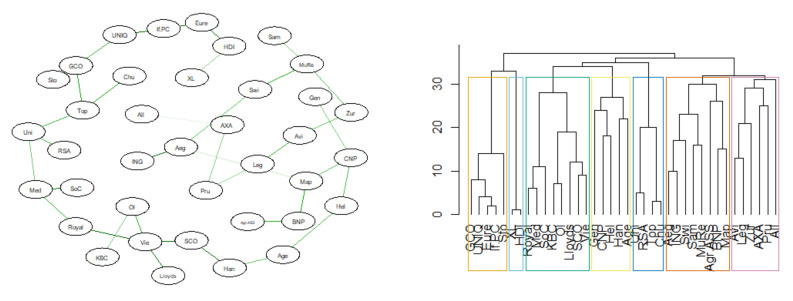
MST based on DTW in I state. Own source.

**Figure 20:**
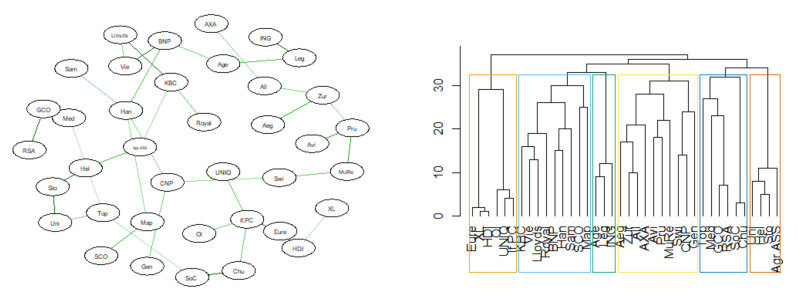
MST based on DTW in FIC state. Own source.

Below are the results of the analysis of the similarity of the DeltaCoVaR time series for all surveyed institutions. The figures (Figure 17, Figure 18, Figure 19 and Figure 20) show the MST in the N-state (Figure 17), SMC (Figure 18), I (Figure 19), and FIC (Figure 20) and the corresponding community structure via greedy optimization of modularity [26]. As a result of this analysis, we found that during the SMC crisis, the MST graph has the most compressed structure, as evidenced by the smallest APL, the largest Max.Degree, and the smallest assortativity (Table 2). The structure of all institutions according to their contribution to the SR, i.e., the DeltaCoVaR measure in the four analyzed periods, shows a division into five groups during the greatest SMC crisis; into six during the FIC crisis in the remaining periods N, which consists of all normal periods; and into seven groups in period I.

In the last part, we present the average values of the topological indices constructed above (Figure 17, Figure 18, Figure 19 and Figure 20) and the MST in subsequent periods. We investigated whether the similarity of DeltaCoVaR in fixed periods between the pairs of the surveyed insurance institutions, and thus the similarity of the contribution to SR, can be assessed on the basis of the topological indexes of MST constructed in a different way than before. A distinctive period is the SMC. APL is smallest, Max.Degree is largest, RCE for k = 2 is largest, and the assortativity value is smallest. The alpha parameter values are not in (2, 3). Thus, it confirms the visual image of MST, on which we observe the lack of the so-called hubs. From the analysis of topological indicators, it can be concluded that the contribution to SR of each institution is at a different level.

## 4. Conclusions

The presented analysis is the first work in the literature in which the possibilities of identifying SR in the insurance sector with the use of a hybrid model are determined by the copula-DCC-GARCH-based MST with the DTW algorithm.

The model was constructed with the use of copula-DCC-GARCH modeling and the innovative MST construction based on dependencies in the tails. Then, we used the DTW algorithm to analyze the similarity in different market regimes time series of the MST topological indicators. The results obtained confirm the possibility of identifying SR in the insurance sector using the presented model. The DTW algorithm we exploited has great potential. Our empirical research confirms the property of the APL indicator known in the literature. APL is the most powerful indicator that describes the structure of MST. In the regimes established, the similarity of the APL indicator series measured using the DTW algorithm was as we expected. We obtained a division into two groups of similar periods: a group of crises and a group of normal periods. For the Diameter indicator, we also obtained two groups. SMC and FIC formed a unique cluster separated from the others. The remaining indicators such as alpha, RCE, and assortativity as interpreted in the context of SR also fulfilled their purpose. The similarity of the dynamics of these indicators was different than in the case of APL, Diameter, and Max.Deg, due to the different roles they play in the MST topology.

The examined topological indicators constructed on the basis of the DTW DeltaCoVaR matrix, MST for pairs of insurance institutions in established market states, also distinguished the SMC crisis, not only because of the level of contribution to the SR, which was due to boxplots, but also because of the dynamics of DeltaCoVaR change over time.

We indicated the further direction of research. It would be sensible to also use the DTW algorithm to identify periods that are similar with respect to SR in the insurance sector, i.e., in the opposite situation than the one studied in this paper. The DTW algorithm should be used to check the similarity of time series of logarithmic rates of return.

We examined the similarity of selected series of MST topological indicators constructed in an innovative way, based on the tail dependence of the logarithmic distributions of weekly returns of 38 insurance companies from the list of the 50 largest systemically significant insurance institutions, determined on the basis of historical data related to financial, economic, and political events in EU countries.

## Figures and Tables

**Table 1:** Standardized DTW. Own source.

	APL	Diameter	Max.Deg	Alpha	RCE	Assortat.	Delta CoVaR
N1-N2	0.1415	0.7691	0.2381	0.2429	0.2486	0.0356	0.0044
N1-N3	0.1670	0.7311	1.2061	0.5678	0.3547	0.1221	0.0087
N1-N4	0.1320	0.6459	0.3688	0.2839	0.3031	0.0353	0.0083
N2-N3	0.1965	0.9689	1.2737	0.5480	0.1914	0.1199	0.0079
N2-N4	0.1741	0.9364	0.3485	0.3119	0.0915	0.0402	0.0050
N3-N4	0.1415	0.6348	1.1138	0.4948	0.1245	0.1129	0.0094
N1-SMC	0.2155	1.0845	0.6240	0.3328	0.3181	0.0348	0.0383
N1-I	0.1677	0.6190	0.3352	0.4661	0.4580	0.0538	0.0099
N1-FIC	0.2264	0.9463	0.3058	0.3995	0.3786	0.0444	0.0156
N2-SMC	0.2073	1.1236	0.6024	0.3600	0.0865	0.0396	0.0408
N2-I	0.1914	0.9087	0.2573	0.4595	0.3183	0.0544	0.0110
N2-FIC	0.2264	1.0082	0.2576	0.4092	0.1642	0.0510	0.0127
N3-SMC	0.2975	1.1755	1.5292	0.4380	0.1815	0.1118	0.0374
N3-I	0.2488	0.7529	1.2595	0.3047	0.2243	0.0893	0.0070
N3-FIC	0.2785	1.0607	1.2063	0.4293	0.0931	0.1024	0.0151
N4-SMC	0.2369	1.2986	0.8855	0.2541	0.0918	0.0374	0.0421
N4-I	0.1774	0.7061	0.3342	0.3600	0.2940	0.0456	0.0141
N4-FIC	0.2500	1.2132	0.2753	0.2658	0.1026	0.0340	0.0082
SMC-I	0.1520	0.9661	0.6336	0.2805	0.2996	0.0509	0.0338
SMC-FIC	0.1467	0.7658	0.6972	0.2686	0.1291	0.0478	0.0436
I-FIC	0.1391	0.8355	0.1803	0.2293	0.2182	0.0487	0.0195

**Table 2:** MST topological indicators determined on the basis of DTW distance between the time series of the DeltaCoVaR measure of all 38 analyzed institutions. Own source.

	N	SMC	I	FIC
APL	7.1778	6.5021	8.6714	8.4211
max.deg	4.0000	7.0000	4.0000	4.0000
alpha	2.0284	3.6861	3.8064	3.8528
RCE (k=2)	0.4267	0.4410	0.2982	0.1428
RCE (k=3)	0.3410	0.0000	0.0000	0.0000
diameter	0.0124	0.0188	0.0178	0.0119
assortativity	−0.3413	−0.4137	−0.2877	−0.2982

## Data Availability

The data are publicly available.
